# Villous Adenocarcinoma of the Duodenum Invading the Ampulla of Vater: Case Report and Review of Literature

**DOI:** 10.1155/1996/32596

**Published:** 1996

**Authors:** Gaetano Catania, Francesco Cardi, Marcello Migliore, Gaetano Romeo

**Affiliations:** Department of Surgery University of Catania Italy

## Abstract

We report a case of villous adenocarcinoma of duodenum arising from the ampulla of Vater with a review of the literature. Although preoperative endoscopic biopsies were performed, no malignancy was identified. Because of the pathological uncertainty we decided to perform a pylorus-preserving pancreatoduodenectomy. Microscopic examination demonstrated glandular dysplasia with aspects of villous adenoma and well differentiated adenocarcinoma. We conclude that both in malignant cases and in cases with uncertain diagnosis a pylorus-preserving pancreatoduodenectomy is the best surgical treatment because it results in better 5 year survival.


*HPB Surgery*, 1996, Vol.10, pp. 105–109
Reprints available directly from the publisher
Photocopying permitted by license only

© 1996 OPA (Overseas Publishers Association)
Amsterdam B.V. Published in The Netherlands
by Harwood Academic Publishers
Printed in Malaysia

### CASE REPORT
### Villous Adenocarcinoma of the Duodenum Invading the Ampulla of Vater Case Report and Review of Literature

GAETANO CATANIA*, FRANCESCO CARDI,
MARCELLO MIGLIORE and GAETANO ROMEO**

Department of Surgery, University of Catania, Italy, Service of Hepato–biliary and pancreatic diseases,
*Associate Professor of Surgical Oncology,
**Professor of Surgery

*(Received 28 October 1993)*

We report a case of villous adenocarcinoma of duodenum arising from the ampulla of Vater with a review of the literature. Although preoperative endoscopic biopsies were performed, no malignancy was identified. Because of the pathological uncertainty we decided to perform a pylorus-preserving pancreatoduodenectomy. Microscopic examination demonstrated glandular dysplasia with aspects of villous adenoma and well differentiated adenocarcinoma. We conclude that both in malignant cases and in cases with uncertain diagnosis a pylorus-preserving pancreatoduodenectomy is the best surgical treatment because it results in better 5 year survival.

KEY WORDS: Villous adenocarcinoma duodenum obstructive jaundice Vater's ampulla

### INTRODUCTION

Villous adenomata of the duodenum represent 45% of all benign lesions of the duodenum, 1% of all duodenal tumors[1] and 2.4% of all benign tumors of the small bowel[2].

This tumor was first described by Perry in 1893 and to date 236 cases have been reported. Recently a high incidence of malignant degeneration has been reported ranging from 35% to 63%[3,4]. Other reports have indicated that the incidence of villous adenoma associated with carcinoma in situ is 16%[3] and that the ampulla of Vater is involved in 47,8%[5] to 82% of cases[3,6–11].

We report a new case of villous adenoma of the duodenum involving the ampulla of Vater and a review of the literature on compare the results of surgical and endoscopic treatment.

*Correspondence to*: Prof. Gaetano Catania, Prof. Ass. di Oncologia Chirurgica, Dipartimento di Chirurgia. Ospedale Vittorio Emanuele, Via Plebiscito 624, 95124 Catania (Italy).

### CASE REPORT

A. 46 year old male presented with a 4 year history of recurrent episodes of jaundice, nausea and intermittent fever that resolve with antibiotic therapy. An ultrasound scan revealed an enlarged liver with dilatation of the gallbladder, pancreatic duct and biliary tree to 2 cm diameter. Endoscopy revealed a tumor invading the second portion of duodenum at the level of the papilla, which on biopsy was a villous adenoma. Endoscopic retrograde cholangio-pancreatography (ERCP) demonstrated a mass invading the duodenum and ampulla of Vater (Fig. 1). A biopsy demonstrated a villous adenoma with moderate dysplasia and positive carcino embryonic antigen (CEA).

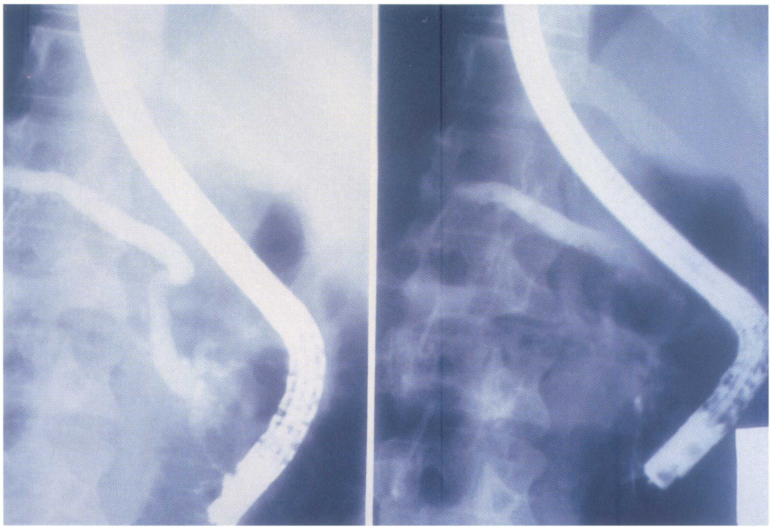

**Figure 1** Endoscopic retrograde cholangio-pancreatography demonstrated a mass invading the duodenum and ampulla of Vater. See Color Plate I.

Blood analyses were all normal except for an alkaline phosphatase of 373 U/I and a white count of 11,500/mm$^3$.

Hypotonic duodenography with contrast revealed a thickening of the wall at the level of the third part of the duodenum. Abdominal CT scan did not identify any enlarged lymphnodes, hepatic or pancreatic lesions.

On the basis of the pathological uncertainty we decide to perform a pylorus preserving pancreatoduodenectomy (PPD).

Gross analysis of the operative specimen showed a plaque lesion 6x4 cm with villi invading circumferentially the pancreatic duct and the common bile duct at the papilla level for 0.8 cm; the duodenal mucosa appearances were of a pseudopolyp (Fig. 2). Microscopic examination demonstrated a glandular dysplasia with aspects of villous adenoma and well differenciated adenocarcinoma. The tumor was predominantly intramucosal with spread in the muscolaris mucosa. Nodes showed only reactive change (Fig. 3). The postoperative period was unremarkable with a hospital stay of 15 days. Three months postoperatively Tc 99 (technetium) scintigraphy showed a prompt visualization of the liver with late images of the ectatic intrahepathic biliary tree. Gastroduodenal manometry demonstrated Motor Migrating Complex (CMM) in the jejunum with the normal presence of phase I after phase II and a longer phase III of Interdegestive Motor Complex (IMC). At two years follow-up the patient is disease free.

### REVIEW OF THE LITERATURE

To December 1991 two hundred-thirty-six cases of villous adenoma of the duodenum have been described. In 148 patients (63%) the tumor involved the ampulla of Vater and are the subject of the study. We excluded 28 patients because lack of information[7], having 120 cases in the study group.

We analyzed several factors including: age, sex, benign or malign pathology, and mean survival after diagnosis.

Statistical analysis was performed using the student *t*-test for unpaired data using a Mystat statistical package running on Apple computer. $p < 0,05$ was considered significant.

Age and sex information was available in 83 patients. Age ranged between 11 and 80 years with a mean of 60.4; mean age of patient with benign tumors was 60.8 and that for malignant 60. Forty-two tumors out these 83 patients were malignant and 41 benign. Of the 42 male patients 25 (60%) had malignant tumors and 17 benign tumors. Of the 41 female patients 24 (58,5%) had benign tumors and 17 malignant.

The histological type of tumour was evaluated in 120 patients, of these 61 were benign and 59 had signs of malignancy.

PPD has been performed in 50 patients. Of these 41 were for malignant tumors and 9 for benign (adenoma with or without cellular atypia). One patient with a benign tumour died post-operatively giving a overall mortality of 1,8%.

Transduodenal or endoscopic excision was performed in 13 malignant and 50 benign cases. Six out of

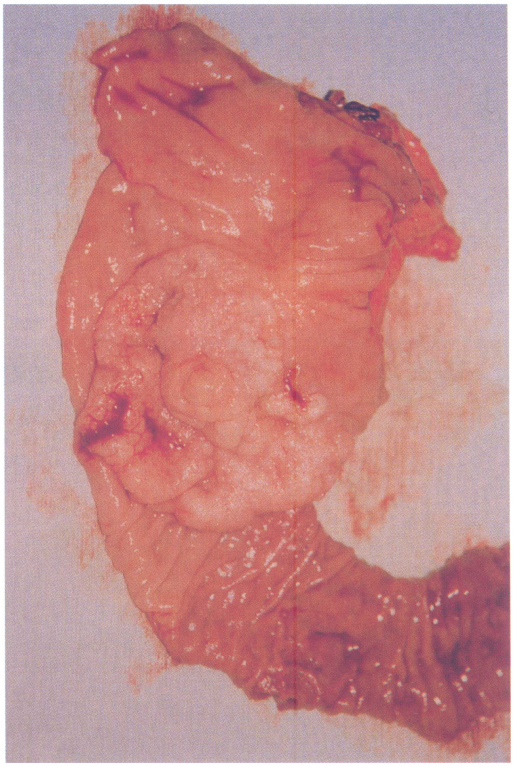

**Figure 2** Pancreaticoduodenectomy: operative specimen showed a plaque lesion with villi invading circumferentially the pancreatic duct and the common bile duct at the papilla. See Color Plate II.

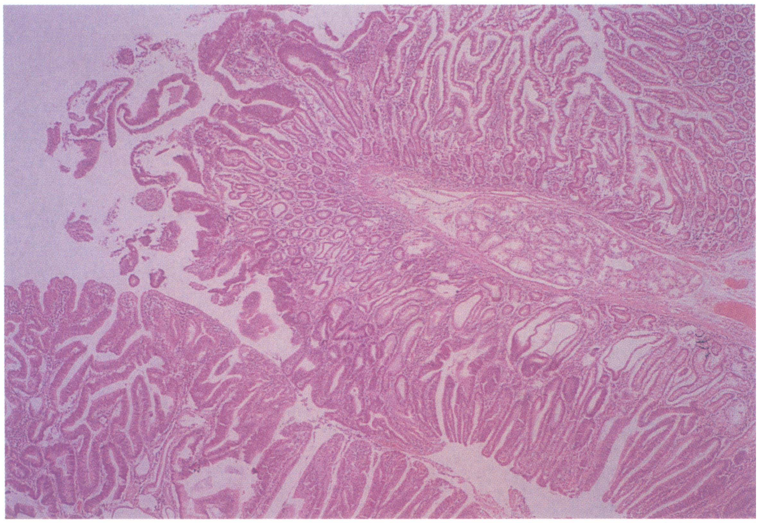

**Figure 3** Photomicrograph of adenocarcinoma of duodenum involving papilla of Vater. The tumor was predominantly intramucosal with spread in the muscolaris mucosa. See Color Plate III.

50 (12%) benign patients presented with a recurrence within 5 years, 1 of these had characteristics of malignancy; PPD was performed in 4, transduodenal in 1 and endoscopic excision in 1 case.

Survival of the 27 patients with malignant tumours who underwent PPD was 52% at 2 years and 22% at 5 years, with a overall mean survival of 44 months.

Eight patients with malignant neoplasia who underwent endoscopic excision had a mean survival of 40 months ($p < 0.05$ versus DCP), 37% at 2 years and 12,5% at 5 years.

Surgical by-pass was performed in 7 patients with a mean survival of 17 months.

### DISCUSSION

Villous adenoma are more frequent in the colon than in the small bowel (40:1)[10]. In the duodenum they are often associated with cholelithiasis[12,13] and familial polyposis.

Our experience shows that the mean age of affected patients in both groups was 60 years; it is also interesting that females have a higher incidence of benign tumors (male:female 0.7) and males have a higher incidence of malignant tumours (male:female 1,47).

The association between carcinoma and villous adenoma of the duodenum has been reported by several authors[14–17]; this is demonstrated by the coesistence of adenoma, residual adenomatosis and microadenoma in 91,4% out of 58 cases of invasivecarcinoma of ampulla of Vater[15] and of residual adenomatosis in 81,8% of ampullary carcinoma[17].

The histological classification often used is that proposed by Komorowski and Cohen[16] who classified carcinoma in situ (CIS) and invasive carcinoma (Inv) as malignant and adenoma with or without cellular atypia as benign.

Diameter, histological type, absence or presence of involved lymphnodes and pancreatic invasion do not have any effect on survival[16,17]. Kutin states that the presence of a villous adenoma larger than 4 cm can be a sign of degeneration[1].

The symptomatology is never obvious. The diagnosis is often made by chance during radiography, endoscopy, duodenal operation for other diseases[18] or autopsy[5]. The most frequent symptoms are: jaundice (69%), abdominal pain (40%) anaemia or melena (19%), weigh loss (18%) and fever (18%). The passing of mucous with consequent loss of electrolytes, described in villous adenoma of colon and rectum, are rarely associated with duodenal adenoma[5].

Endoscopy is the best procedure to identified villous adenoma because of the opportunity for biopsy but unfortunately the occurrence of false negative is of the order of 33% because of the small dimension of the biopsies; this problem is often encountered during intraoperative biopsies[5]. Endoscopy can also be used in particular cases to decompress the biliary tree by sphincterotomy or by positioning a bile drainage catheter to avoid infection and complications such as haemorrhahage and postoperative renal failure[19].

Ultrasonography rarely has been useful[5] even if a "pseudokidney" has been identified[20]. Recently the utilization of echoendoscopy permits more accurate identification of a villous adenoma with malignant degeneration invading submucosal layers[21].

Hypotonic duodenography, can help to identify the lesion that occurs in the second portion of the duodenum. CT scan of the upper abdomen is useful to show any sign of local spread and nodal involvement.

An open problem is the treatment that can be surgical (PPD or transduodenal excision), endoscopical or in the inoperable cases a palliative by pass.

Chappuis[5] states that endoscopic excision has to be performed in all cases except those with invasive carcinoma. Evans[22] believes that endoscopic exision should be confined to a polypoid lesion. Some authors think that local excision should be reserved for villous adenoma with carcinoma in situ[5,12], others believe this should be reserved for tumours of the I, II and IV duodenal portion[4,22].

Surgical or endoscopic excision produces an incidence of recurrence of 28% with a disease free mean of 12 months[6], also if the tumor involves the lumen of the biliary or pancreatic tree than a higher incidence of haemorrhahage, perforation or recurrence will be found[22]. Our study shows a post-operative recurrence rate for benign villous adenoma of 12,5%. Unfortunately the recurrence is not always benign. One patient presented with malignant degeneration in the recurrence.

There are several indications for PPD: a) invasive carcinoma[5,12,23], b) invasion of ampulla of Vater[6], c) cancer clearance (operative mortality of 5%, 5 year survival 30–50%).

Because of the possibility of a false negative biopsy in carcinoma, we think that PPD should also be performed in those patients with uncertain preoperative diagnosis; PPD is also indicated for recurrent tumours with possible degeneration. We also believe that a surgeon must be able intraoperatively to change, on the basis of the local situation (dimension, nodes involvement, and histologic responce), the surgical excision to the more complex PPD. Duodeno-pancreatectomy is associated with a overall mortality of 2%.

Our review shows that the mean survival after PPD differ statisticaly compared with local excision; in particular the 2 year survival is higher in patients who underwent PPD (52%) versus 37% in patients who only had excision of the tumour. The 5 years survival is 22% in the PPD group versus 12% in the excision group. These results in our opinion, suggest that PPD is a better operation for these tumours and is associated with a better 5 years survival.

Surgical by-pass has the lowest mean survival because this procedure is always performed for advanced tumour.

### CONCLUSION

Diagnosis is often difficult on endoscopy even when examination is associated with a biopsy and echoendoscopy.

In obvious benign cases the choice is between the endoscopic excision (polypoid and small tumors) or trans-duodenal excision.

In malignant cases pylorus preserving pancreatoduodenectomy is the best surgical treatment because it results in better 5 year survival.

If the diagnosis is uncertain than a PPD is to indicated because the possibility of a occult carcinoma.

### ACKNOWLEDGEMENTS

The authors thank Mr. Maxwell Murison, senior register plastic surgery at Ulster Hospital in Belfast (Ireland), for reviewing this manuscript and Dr. Giovanna Russo, pathologist, for photomicrography.

### REFERENCES

1. Kutin, N. D., Ranson, J. H., Gouge, T. H. and Localio, S. A. (1975) Villous Tumors of the Duodenum. *Ann. Surg*, **181**, 164–168.
2. Wilson, J. M. Melvin, B. D., Gray, G. and Thorbjarnarson, B. (1975) Benign Small Bowel Tumor. *Ann. Surg*, **181**, 247–250.
3. Ryan, D. P., Schapiro, R. H. and Warshaw, A. L. (1986) Villous Tumors of the Duodenum. *Ann. Surg*, **203**, 301–306.
4. Schulten, M. F. Jr., Oyasu, R. and Beal, J. M. (1976) Villous adenoma of the duodenum. A Case Report and Review of the Literature. *Am. J. Surg.*, **132**, 90–96.
5. Chappuis, C. W., Divincenti, F. C. and Cohn, I. (1989) Villous Tumors of the Duodenum. *Ann. Surg*. **209**, 593–599.
6. Bjork, K. J., Davis, C. J., Nagorney, D. M. and Mucha, P. Jr (1990) Duodenal Villous Tumors. *Arch. Surg*. **125**, 961–965.
7. Galandiuk, S., Hermann, R. E., Jagelman, D. G., Fazio, V. W. and Sivak, M. V. (1988) Villous Tumors of the Duodenum. *Ann. Surg*. **207**, 234–239.
8. Krukowski, Z. H., Ewen, S. W. B., Davidson, A. I. and Matheson, N. A. (1988) Operative management of tubulovillous neoplasms of the duodenum and ampulla. *Br. J. Surg*. **75**, 150–153.
9. Geier, G. E., Gashti E. N., Houin, H. P., Johnloz, D. and Madura, J. A. (1984) Villous adenoma of the duodenum: clinicopathologic study of five cases. *Am. Surg*. **50**, 617–622.
10. Celik, C., Vendetti, J. A. Jr. Satchidanand, S. *et al.* (1986) Villous tumors of the duodenum and ampulla of Vater. *J. Surg. Oncol*. **33**, 268–272.
11. Thorbeck, C. V., and Martinez, M. S. (1989) Villous Adenoma of the duodenum. Case Report. *Acta Chir. Scand*. **155**, 71–73.
12. Rosemberg, J., Welch, J. P., Pyrtek, L. J., Walker M. and Trowbridge P. (1986) Benign villous adenomas of the ampolla di Vater. *Cancer* **58**, 1563–1568.
13. Oh, C., Jemerin, E. E. (1965) Benign adenomatous polyps of the papilla of Vater. *Surgery* **57**, 495–503.
14. Perzin, K. H. and Bridge, M. F. (1981) Adenomas of the Small Intestine. A Clinicopathologic Review of 51 Cases and a Study of Their Relationship to Carcinoma. *Cancer* **48**, 799–819.
15. Baczako, K., Buchler, M., Beger, H., Kirkpatrick, J. and Haferkamp, O. (1985) Morphogenesis and possible precursor lesions of invasive carcinoma of the papilla of Vater: Epithelial dysplasia and adenoma. *Hum. Pathol*. **16**, 305–310.
16. Komorowski, R. A. and Cohen, E. B. (1981) Villous tumors of the duodenum. A clinicopathologic study. *Cancer* **47**, 1377–86.
17. Kozuka, S., Tsubone, M., Yamaguchi, A. and Hachisuka, K. (1981) Adenomatous residue in cancerous papilla di Vater. *Gut* **22**, 1031–34.
18. Noya, G., Dettori, G., Soro, P., Niolu, P., Muscas, A., Marongiu, G. and Biglioli, P. (1983) L'adenoma villoso del duodeno. *Min. Chir*. **38**, 1357–1360.
19. Alderson, D., Lavelle, M. I. and Venables, C. W. (1981) Endoscopic sphincterotomy before pancreaticoduodenectomy for ampullary carcinoma. *Brit. Med. J*. **282**, 1109–1111.
20. Bluth, E. I., Merrit, C. R. B. and Sullivan, M. A. (1979) Ultrasonic evaluaiton of the stomach, small bowel, and colon. *Radiology* **133**, 677–680.
21. Tio, T. L., Tytgat, G. N. J. (1989) Endoscopic Ultrasonography in the operative staging of Biliopancreatic Carcinoma In *Hepatobiliary and Pancretic Malignancies. Diagnosis, Medical and Surgical Management*, edited by N.J. Lygidakis and G.N.J. Tytgat, pp. 66–78. George Thieme Verlag Stuttgart, New York.
22. Evans, P. R., Oliver, D. J., Waters, S. A., Waters, T. E., Lawrence-Brown, M. M. D. and Easton, L. A. (1990) Villous adenomas of the duodenum and an unusual variant. *Austr. N.Z. J. Surg*. **60**, 887–892.
23. Haglund, U., Fork, F. T., Genell, S. and Rehnberg, O. (1985) Villous adenomas in the duodenum. *Br. J. Surg*. **72**, 26–27.

